# Prospective study on diagnostic efficacy of targeted NGS vs. traditional testing for respiratory infections in myelosuppressed hematology patients

**DOI:** 10.3389/fmed.2025.1488652

**Published:** 2025-03-12

**Authors:** Chen Li, Jing Wu, Yuhu Feng

**Affiliations:** ^1^Department of Hematology, Fuyang People’s Hospital (The Affiliated Fuyang People’s Hospital of Anhui Medical University), Fuyang, China; ^2^Department of Hematology, The Second Affiliated Hospital of Chengdu Medical College, China National Nuclear Corporation Hospital 416, Chengdu, China

**Keywords:** tNGS, hematologic malignancies, respiratory infections, pathogen detection, immunocompromised patients, traditional microbiological testing, antimicrobial therapy

## Abstract

**Background:**

Respiratory tract infections are a significant complication in myelosuppressed hematological patients, especially those with myelosuppression. Traditional microbiological testing methods often show limitations in sensitivity, turnaround time, and cost, making them less effective in this vulnerable population. This study aimed to evaluate the diagnostic efficacy of targeted next-generation sequencing (tNGS) compared to traditional microbiological testing methods (TMT) in detecting respiratory infections among myelosuppressed hematological patients.

**Methods:**

This prospective study included 20 patients aged ≥15 years with myelosuppressed hematological disease and respiratory infection, admitted to the hematology department of Fuyang People’s Hospital between January and May 2024. Eligible patients underwent both 198-pathogen respiratory tract infection targeted NGS panel (198-pathogen RTI tNGS panel) and TMT. Exclusion criteria included refusal of tNGS or incomplete sputum collection. The diagnostic performance of 198-pathogen RTI tNGS panel was assessed against TMT, with diagnoses confirmed by three independent hematology experts. The study adhered to ethical guidelines and obtained informed consent from all participants.

**Results:**

tNGS demonstrated a 100% pathogen detection rate compared to 40% with traditional testing (*p* < 0.001). It identified a broader spectrum of pathogens, including bacteria, viruses, and fungi, many of which were missed by TMT. The most common pathogens isolated in the clinical specimens detected by TMT was *Epstein–Barr virus*. The most common pathogens isolated in the clinical specimens detected by 198-pathogen RTI tNGS was *novel coronavirus*, *human respiratory syncytial virus type B*, and *influenza A virus*. The sensitivity of tNGS was 94.74%, with a positive predictive value of 100%. The turnaround time for tNGS was significantly shorter, averaging 24 h, enabling quicker adjustments to antimicrobial therapy. In 75% of cases, the tNGS results directly influenced changes in treatment regimens, improving clinical outcomes.

**Conclusion:**

tNGS offers superior sensitivity, a broader pathogen detection range, and a faster turnaround time compared to traditional microbiological testing methods. It provides a practical and efficient diagnostic option for respiratory infections in hematological patients, particularly those unable to undergo invasive procedures such as bronchoalveolar lavage. The use of tNGS may enhance clinical management and improve patient outcomes in this population.

## 1 Introduction

In patients with myelosuppressed hematological diseases, respiratory tract infections are a common and serious complication, particularly in those with agranulocytosis (neutrophil count <0.5 × 10^9^/L) and fever. These patients require prompt treatment and accurate diagnosis, but are often unable to undergo tracheal lavage due to thrombocytopenia and can only undergo tests based on sputum and blood samples. Traditional microbiological tests (TMT), including general bacterial culture, blood culture, fungal culture, fungal smear, respiratory six-pathogen detection (virus), and SARS-CoV-2 nucleic acid testing, have limitations such as low sensitivity (1, 2), limited specimen types, long turnaround times, high economic burden, and unidentified infections. Metagenomic NGS improves detection but is costly (3–6). On the other hand, targeted next-generation sequencing (tNGS), in combination with ultra-polymerase chain reaction (PCR) and high-throughput sequencing technology allows for simultaneous identification of hundreds or thousands of common pathogens (7, 8). Several studies have demonstrated the effectiveness of tNGS in detecting respiratory pathogens at a quarter of the cost of mNGS (7). Another study showed that microbiological tests based on bronchoalveolar lavage fluid had similar diagnostic performance between tNGS and mNGS (8). Sputum pathogen-targeted high-throughput sequencing (tNGS) provides a more rapid, convenient, efficient and inexpensive pathogen detection method compared with traditional microbiological tests. It has been utilized for diagnosing challenging infectious diseases. Some studies have confirmed that sputum-based tNGS demonstrates good diagnostic efficiency for patients with respiratory tract infections (6). However, these findings did not include patients with hematological diseases. Patients with hematological diseases often cannot undergo bronchoalveolar lavage, and routine sputum microbial detection has a low positive rate. However, sputum-based tNGS has a higher positivity rate and detects resistant genes that may be used to diagnose respiratory infections (9). Retrospective studies comparing tNGS to traditional testing in patients with hematological diseases and respiratory infections are lacking. Therefore, this study used an updated 198-pathogen respiratory tract Infection targeted NGS panel to analyze pathogen distribution and clinical decision-making (Supplementary Table S1, for scholarly reference only; commercial use is prohibited) in patients with hematological diseases.

## 2 Materials and methods

### 2.1 Study subjects and methods

**Figure 1:**
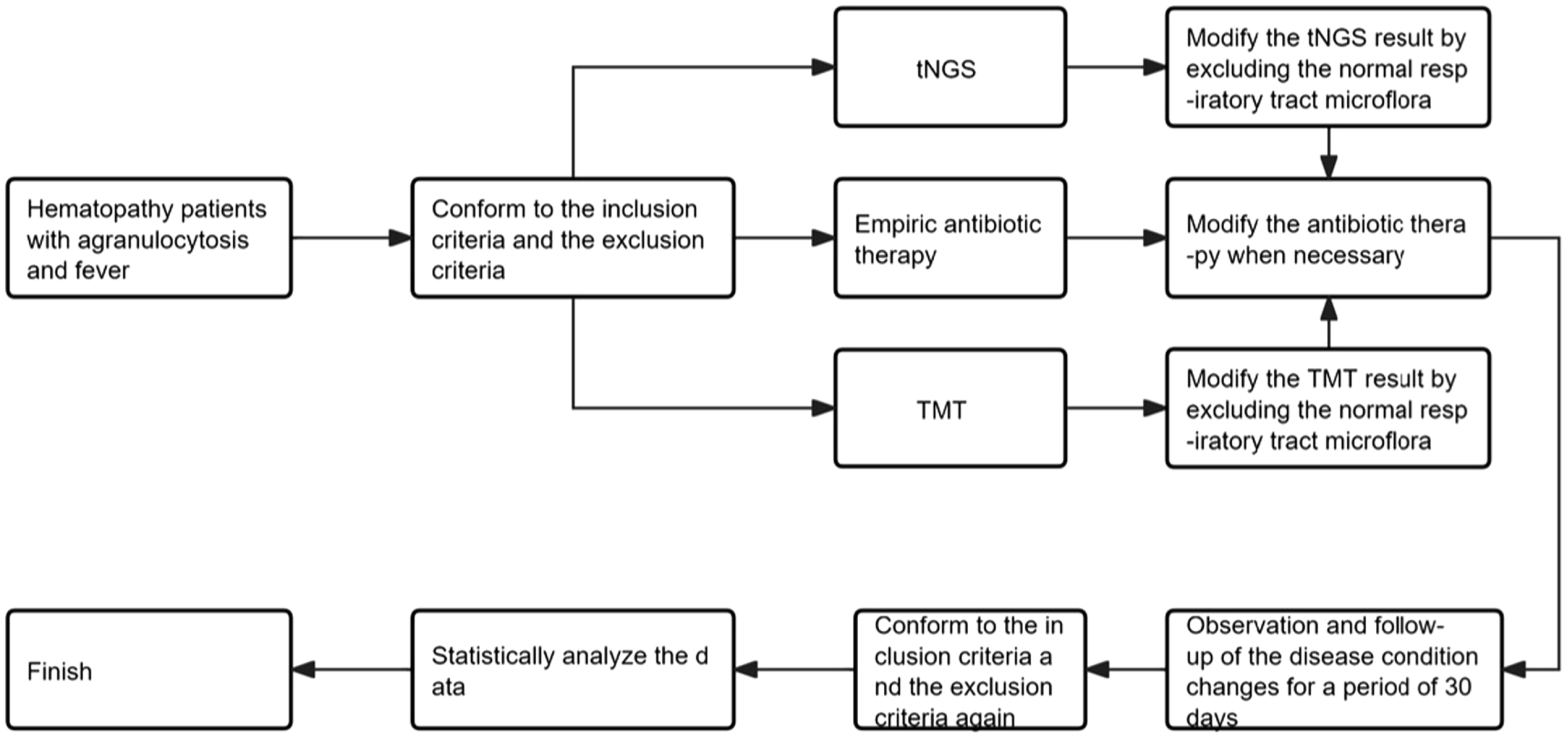
Flowchart of the clinical study.

The flowchart of this study is shown in Figure 1.

The eligibility criteria for this study included the following: (1) admission to the hematology department of Fuyang People’s Hospital from January to May 2024. (2) Age ≥15 years old. (3) Provided informed consent. (4) With myelosuppressed hematological disease. (5) With respiratory infection. (6) Underwent both 198-pathogen RTI tNGS panel and CMT. The exclusion criteria included the following: (1) refused 198-pathogen RTI tNGS panel. (2) Did not comply with the instructions for sputum collection. (3) The medical record data is incomplete. Results of 198-pathogen RTI tNGS panel, general bacterial culture, blood culture, fungal culture, fungal smear examination, respiratory six-pathogen detection (virus), SARS-CoV-2 nucleic acid detection, imaging findings, clinical symptoms, medical history, and treatment response for each enrolled patient were obtained. Follow-up was conducted until 14 days after hospital discharge or death (whichever occurred first) for all enrolled patients. All enrolled patients adhered to the sputum collection requirements (2). Nurses instructed patients to brush their teeth in the morning, rinse with saline, perform deep breathing exercises, and then forcefully expectorate sputum from the respiratory tract, taking care to avoid contamination with oral and nasopharyngeal secretions. Samples were collected in sterile containers with lids (2). Patients were explicitly informed that they should cough forcefully to expectorate sputum and should not collect saliva in the container. Enrollment was voluntary and safety-related data were assessed first during the study. Recruitment was stopped if any safety concerns occurred. The study was approved by the Ethics Committee of Fuyang People’s Hospital [Approval Number: Medical Ethics Review (2024)96] and complied with the Declaration of Helsinki guidelines. All patient data used in this study were obtained anonymously for exclusive use in this paper analysis while strictly protecting patient privacy rights; informed consent was obtained from all patients involved.

### 2.2 Detailed information about the detection methods

#### 2.2.1 198-pathogen RTI tNGS panel

Targeted next-generation sequencing (tNGS) combines ultra-polymerase chain reaction (PCR) and high-throughput sequencing technology. The test sample is the patient’s sputum. In this study, we employed an expanded pathogen spectrum, covering 198 common respiratory pathogens—significantly more than the 98–158 pathogens typically included in previous research. This comprehensive spectrum includes bacteria (encompassing *Mycobacterium tuberculosis* and non-tuberculous mycobacteria), fungi, viruses, mycoplasma, chlamydia, *Coxiella burnetii*, and common pathogen resistance genes, which together account for over 98% of pathogens responsible for respiratory infections. This panel is referred to as 198-pathogen RTI tNGS panel (For scholarly reference only; commercial use is prohibited Supplementary Table S1) (2). The following outlines the detailed steps involved.

##### 2.2.1.1 Sample preparation

To prepare the sample, 650 μL was combined with an equal volume of 80 mmol/L dithiothreitol in a 1.5 mL centrifuge tube, and the mixture was homogenized for 15 s using a vortex mixer. Additionally, both a positive and a negative control from the Respiratory Pathogen Detection Kit (KS608-100HXD96, KingCreate, Guangzhou, China) were included to oversee the entire targeted next-generation sequencing (tNGS) procedure.

##### 2.2.1.2 Nucleic acid extraction

Following homogenization, 500 μL of the sample mixture was used for the extraction and purification of total nucleic acids. This was performed using the MagPure Pathogen DNA/RNA Kit (R6672-01B, Magen, Guangzhou, China), adhering to the manufacturer’s instructions.

##### 2.2.1.3 Library construction and sequencing

The sequencing library was constructed with the Respiratory Pathogen Detection Kit, and a no-template control was incorporated to track both the library construction and sequencing stages. This procedure included two rounds of polymerase chain reaction (PCR) amplification. Nucleic acids from the sample and complementary DNA (cDNA) served as templates, with a selection of 153 microorganism-specific primers employed for ultra-multiplex PCR. This approach enriched the target pathogen sequences, covering a range of pathogens such as bacteria, viruses, fungi, mycoplasma, and chlamydia. After amplification, the PCR products were purified using beads, then subjected to a secondary amplification with primers containing sequencing adapters and unique barcodes. The quality and quantity of the prepared library were assessed using a Qsep100 Bio-Fragment Analyzer (Bioptic, Taiwan, China) and a Qubit 4.0 fluorometer (Thermo Scientific, Massachusetts, United States), respectively. Typically, the fragment size of the library was between 250 and 350 bp, with the library concentration being maintained at or above 0.5 ng/μL. The concentration of the final mixed library was re-evaluated and diluted to a final concentration of 1 nmol/L. Then, 5 μL of this mixed library was combined with 5 μL of freshly prepared 0.1 mol/L NaOH, vortexed briefly, and centrifuged. The denatured library was incubated at room temperature for 5 min, then subjected to sequencing using the Illumina MiniSeq platform with a universal sequencing reagent kit (KS107-CXR, KingCreate, Guangzhou, China). On average, each library generated approximately 0.1 million reads with a single-end read length of 100 bp.

##### 2.2.1.4 Bioinformatics analysis

The sequencing data were processed using the data management and analysis system (v3.7.2, KingCreate). The raw data were initially processed for adapter identification, retaining reads with single-end lengths greater than 50 bp. Low-quality reads were filtered, and those with a Q30 score above 75% were selected for further analysis to ensure high data quality. The filtered, single-ended reads were then aligned to a self-building clinical pathogen database, and the read count for specific amplified targets was determined for each sample. Reference sequences for read mapping were sourced from various databases, including GenBank, RefSeq, and the NCBI Nucleotide database (2).

##### 2.2.1.5 Expert-confirmed pathogen identification

The results of 198-pathogen RTI tNGS panel test may include normal colonizing bacteria of the respiratory tract, which will be noted in the report. The pathogenic microorganisms were identified by hematology specialists based on the pathogenicity of different organisms in relation to the patient’s imaging findings, clinical symptoms, medical history, and treatment response. The diagnosis was confirmed by three independent hematology experts.

#### 2.2.2 TMT

Traditional microbiological tests (TMT), including general bacterial culture, blood culture, fungal culture, fungal smear, respiratory six-pathogen detection (virus), and SARS-CoV-2 nucleic acid testing. The test sample of general bacterial culture, fungal culture, fungal smear, respiratory six-pathogen detection (virus), and SARS-CoV-2 nucleic acid testing is the patient’s sputum. The test sample of blood culture is the patient’s blood. The TMT can detect bacteria, fungi, and seven types of viruses. However, certain viruses, such as Epstein–Barr virus (EBV), cannot be detected by TMT but can be identified through tNGS. This highlights the superiority of TMT. Each detection method has its own specific procedure. General bacterial culture involves collecting sputum and inoculating them onto solid or liquid media that support the growth of bacteria. The samples are incubated at optimal temperatures (typically 35–37°C), and after 24–48 h, bacterial colonies are identified based on morphology, biochemical tests (e.g., Gram stain, catalase test), and molecular methods if necessary. Antibiotic sensitivity testing is often performed to determine the resistance profiles of isolated bacterial strains. Blood culture is positive when bacteria enter the bloodstream in patients with respiratory tract infections. Blood samples are drawn from two separate sites and added to special culture bottles containing nutrient broth. These bottles are incubated in an automated system that detects microbial growth based on changes in CO₂ production or other indicators. Once growth is detected, subcultures are performed for bacterial identification and antibiotic susceptibility testing. For fungal culture, sputum are inoculated onto selective media like Sabouraud Dextrose Agar, which supports the growth of fungi. The samples are incubated at appropriate temperatures (typically 25–30°C for yeasts or 35–37°C for molds) for several days, and fungal species are identified based on colony morphology and microscopic examination of the fungal structures. Antifungal susceptibility testing may also be performed in cases of systemic fungal infections. Fungal smear preparation involves taking sputum and applying it to a microscope slide. After staining with potassium hydroxide (KOH) or Gram stain, the slide is examined under a microscope for the presence of fungal elements, such as hyphae or yeast cells. The respiratory six-pathogen detection (virus) method is a molecular diagnostic technique designed to rapidly identify six common viral pathogens responsible for respiratory infections. This test typically targets influenza A and B viruses, respiratory syncytial virus (RSV), human metapneumovirus (HMPV), adenovirus, and parainfluenza virus. Sputum samples are processed using multiplex PCR to amplify viral RNA, which is then converted into complementary DNA (cDNA). Specific primers for each virus are used in the PCR reaction, and fluorescence-based detection identifies the presence of each virus based on its unique genetic sequence. This provides rapid and accurate results for respiratory infections. Finally, SARS-CoV-2 nucleic acid testing uses RT-PCR to detect the presence of SARS-CoV-2, the virus responsible for COVID-19. A nasopharyngeal or oropharyngeal swab is collected and processed to extract viral RNA. This RNA is then reverse-transcribed into complementary DNA (cDNA) and amplified using PCR primers specific to the SARS-CoV-2 genome, such as the spike protein or nucleocapsid gene. The amplification process is monitored using fluorescence or other detection methods to confirm the presence of the virus.

### 2.3 Evaluation indicators

The study primarily aimed to assess the diagnostic performance of the 198-pathogen RTI tNGS panel test for respiratory tract infections. The secondary objective was to compare the performance of 198-pathogen RTI tNGS panel with TMT and evaluate positive and negative consistency rates and their clinical significance. Positive concordance was defined when 198-pathogen RTI tNGS panel identified at least one of the pathogens when compared to TMT. The positive concordance rate was the number of positive concordances from 198-pathogen RTI tNGS panel divided by those from TMT (10).

Respiratory tract infections were diagnosed by hematologists based on bacterial, blood, and fungal cultures, fungal smears, viral detection (including COVID-19 nucleic acid testing), 198-pathogen RTI tNGS panel results, imaging, medical history, and clinical symptoms, with diagnoses confirmed by three independent experts. The results of TMT and tNGS may include normal colonizing bacteria of the respiratory tract, which will be noted in the report. The pathogenic microorganisms were identified by hematology specialists based on the pathogenicity of different organisms in relation to the patient’s imaging findings, clinical symptoms, medical history, and treatment response. The diagnosis was confirmed by three independent hematology experts. These experts also determined whether antibiotics were adjusted based on the 198-pathogen RTI tNGS panel results and evaluated the effectiveness of the modified antibiotic regimen.

### 2.4 Statistical analysis

Continuous variables are summarized using observations, mean, median, standard deviation, min, and max. Categorical variables are presented as frequency and percentage per category. Data were entered into SPSS 20.0 and R for processing. Measurement data were expressed as (x ± s) and were analyzed by the *t*-test. Count data are expressed as (*n*, %) and compared using the chi-square test. *p* < 0.05 was considered significant.

## 3 Results

### 3.1 Patient characteristics

Overall, 20,198-pathogen RTI tNGS panel test results from 20 patients with hematological diseases were analyzed. The median age of patients was 63 years, and 60% were men. Agranulocytosis and platelet counts <20 × 10^9^ were observed in all patients. Consequently, all patients were unable to undergo bronchoalveolar lavage fluid examination due to their platelet counts (Supplementary Table S3).

### 3.2 Pathogen detection using 198-pathogen RTI tNGS panel and TMT

**Figure 2:**
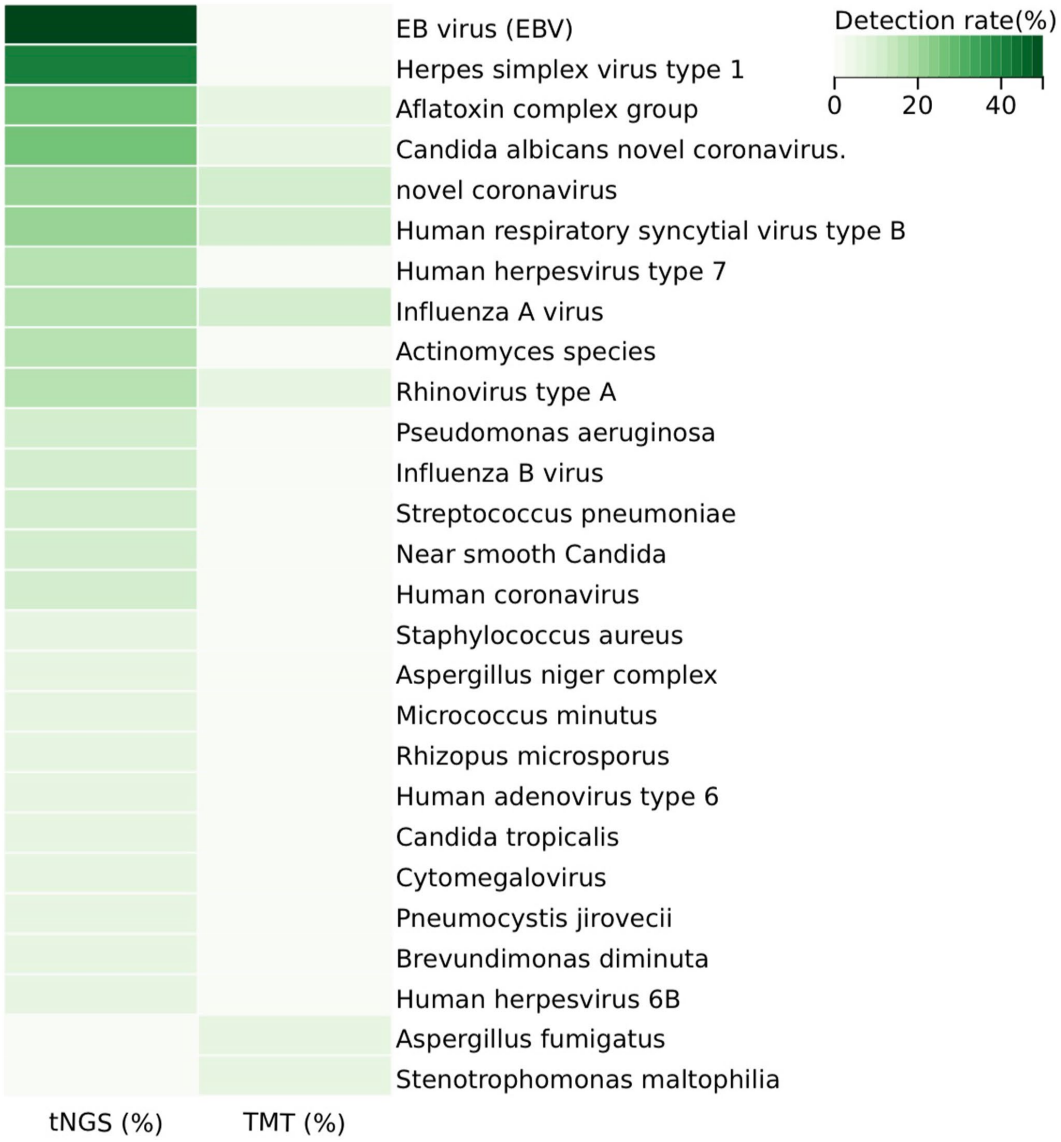
Distribution of pathogens in the study cohort and detection rate heterogeneity between tNGS and TMT. 198#tNGS identified bacteria in 25% of samples, with *Fusobacterium nucleatum* being the most common, while TMT detected bacteria in only 5%. For viruses, 198#tNGS detected 85% of samples, including Epstein–Barr virus (50%), compared to 30% by TMT. Fungal infections were detected in 65% of samples by 198#tNGS, with *Monilia albicans* being the most prevalent, whereas TMT identified fungi in only 10%. This demonstrates the superior sensitivity of 198#tNGS across all pathogen types.

In the sputum samples from 20 patients, the 198-pathogen RTI tNGS panel identified 23 pathogens, whereas the TMT detected eight pathogens (Figure 2 and Supplementary Table S2). Specifically, the 198-pathogen RTI tNGS panel detected the following pathogens: EB virus (EBV), herpes simplex virus type 1, aflatoxin complex group, *Candida albicans*, novel coronavirus, human respiratory syncytial virus type B, human herpesvirus type 7, influenza A virus, *Actinomyces* species, rhinovirus type A, *Pseudomonas aeruginosa*, influenza B virus, *Streptococcus pneumoniae*, near smooth *Candida*, human coronavirus, *Staphylococcus aureus*, *Aspergillus niger* complex, *Micrococcus minutus*, *Rhizopus microsporus*, human adenovirus type 6, *Candida tropicalis*, cytomegalovirus, *Pneumocystis jirovecii*, *Brevundimonas diminuta*, human herpesvirus 6B, *Aspergillus fumigatus*, *Stenotrophomonas maltophilia*. In comparison, the TMT detected the following pathogens: aflatoxin complex group, *Candida albicans* novel coronavirus, rhinovirus type A, *Aspergillus fumigatus*, *Stenotrophomonas maltophilia*, novel coronavirus, human respiratory syncytial virus type B, influenza A virus (Supplementary Table S5).

**Figure 3:**
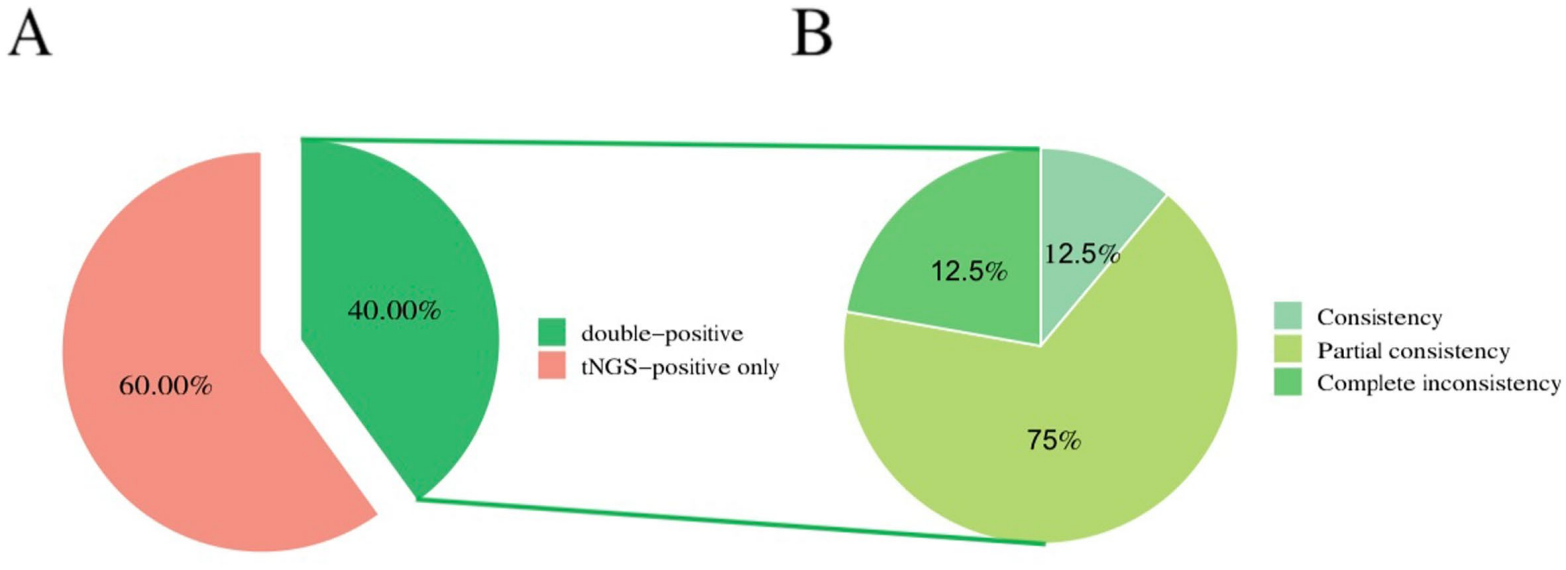
Consistency of pathogen detection between tNGS and CMTs. **(A)** 40% (8/20) of samples were positive on both 198#tNGS and TMT, while 60% (12/20) were positive only on 198#tNGS. No samples were positive only on TMT, and no samples were negative on both methods. **(B)** Among the eight double-positive samples, one showed complete agreement (12.5%, 1/8), six showed partial agreement (75%, 6/8), and one showed complete disagreement (12.5%, 1/8) between the two methods.

The overall microbiological detection rates for 198-pathogen RTI tNGS panel and TMT were 100% (20/20) and 40% (8/20), respectively (*p* < 0.001). The sensitivity, specificity, positive predictive value, and negative predictive value of tNGS were 94.74, 100, 100, and 50%, respectively. Furthermore, 40% (8/20) of samples were positive on both 198-pathogen RTI tNGS panel and TMT, 0% (0/20) were negative on both 198-pathogen RTI tNGS panel and TMT, and 60% (12/20) were positive only on 198-pathogen RTI tNGS panel. Meanwhile, 0% (0/20) were positive on TMT only. Of the eight double-positive samples, one showed complete agreement between 198-pathogen RTI tNGS panel and TMT, six showed partial agreement, and one showed complete disagreement (Figure 3). Moreover, the positive consistency rate of tNGS and blood culture was 87.5%. Common pathogens of respiratory tract infections include bacteria, viruses, fungi, and atypical pathogens. Specifically, 198-pathogen RTI tNGS panel identified bacteria in five samples (25%). The most common bacterium detected was *Fusobacterium nucleatum*, accounting for 15% (3/20) of the total positive detections by 198-pathogen RTI tNGS panel, followed by *Pseudomonas aeruginosa* and *Streptococcus pneumoniae*. In contrast, TMT only detected bacterial infection in one sample (1/20, 5%). Additionally, 198-pathogen RTI tNGS panel identified viruses in 17 samples (85%), with the primary viruses being Epstein–Barr virus (50%), herpes simplex virus type 1, and 2019-nCoV. In contrast, TMT only detected viruses in six samples (30%). Furthermore, 198-pathogen RTI tNGS panel detected fungal infections in 13 samples (65%), with the most prevalent being *Monilia albicans* (25%), followed by *Candida parapsilosis* and *Rhizopus microsporus*. Conversely, TMT only detected fungal infections in two samples (10%).

### 3.3 Clinical implications of 198-pathogen RTI tNGS panel

198-pathogen RTI tNGS panel provides valuable guidance for clinicians’ treatment decisions. Based on the results from 198-pathogen RTI tNGS panel, doctors can combine the patient’s imaging findings, clinical symptoms, medical history and treatment response to identify the pathogenic microorganism and directly guide the selection of the most appropriate antibiotic. The results of 198-pathogen RTI tNGS panel directly influenced antibiotic treatment decisions for 12 patients, including those with *SARS-CoV-2 infection*, *Pneumocystis jirovecii*, and *Stenotrophomonas maltophilia*. Notably, the detection of the methicillin-resistant staphylococcal resistance gene mecA impacted antibiotic treatment decisions for a patient (Patient ID-2, Supplementary Table S5). In contrast to general bacterial culture methods that typically require 3–5 days for results turnaround time (TAT), 198-pathogen RTI tNGS panel demonstrated an average TAT of 24 h. This rapid TAT is significantly shorter than traditional culture methods such as blood culture and fungal culture. Consequently, this highlights that compared to TMT, 198-pathogen RTI tNGS panel offers a faster and more consistent TAT which is crucial in guiding clinical practice by enabling timely adjustments to antibiotic applications based on accurate pathogen identification.

## 4 Discussion

Patients with hematological diseases have an impaired immune system due to the disease itself and subsequent treatments. Respiratory infections in these patients are unique, with higher rates of fungal and atypical pathogens that are often drug-resistant and manifest with severe symptoms (11). As these patients often have a rapidly progressive disease, bronchoscopy is required (12, 13). However, bronchoscopy is not feasible in many patients due to (1) low platelet counts, (2) reduced oxygen saturation due to rapid disease progression, and (3) fear of hemoptysis and bronchoscopy. In this retrospective study, 198-pathogen RTI tNGS panel is highly sensitive and predictive for respiratory infections, faster than other methods, and cost-effective (Supplementary Table S4). Sputum-based 198-pathogen RTI tNGS panel also has high diagnostic efficiency, making it the optimal choice for patients with hematological disease and respiratory infections. tNGS effectively identifies non-bacterial pathogens that are often missed by traditional methods. Additionally, tNGS guides clinicians on the appropriate antibiotics, thus improving patients’ quality of life and survival. Based on the 198-pathogen RTI tNGS panel results in our study, treatment in more than half of patients was adjusted accordingly. Therefore, for patients with hematological diseases and respiratory tract infections who cannot undergo bronchoscopy, 198-pathogen RTI tNGS panel may be a practical option.

While this study provides valuable insights into the application of the 198-pathogen RTI tNGS panel for diagnosing respiratory infections in hematology patients, several limitations must be acknowledged. (1) Sample size: the sample size in this study was limited, and larger studies are planned for the future. However, the 198-pathogen RTI tNGS panel developed in this research addresses the clinical needs of many hematology patients with respiratory tract infections who are unable to undergo bronchoalveolar lavage fluid collection. Despite the limited sample size, this study serves as a pioneering effort in the field of hematological diseases and respiratory infections, retaining significant research value. Larger-scale studies in the future will be crucial to validate its diagnostic capabilities. (2) Sputum liquefaction test: the sputum liquefaction test primarily reflects the predominant pathogens of upper respiratory tract infections. In this study, sputum specimens were the only viable option for patients with respiratory tract infections who were unsuitable for bronchoalveolar lavage fluid testing. This unique clinical context led to our retrospective study, which aimed to utilize tNGS to improve the positive detection rate of sputum specimens and guide clinical decisions. To mitigate the risk of contamination, patients adhered to strict sputum collection protocols, which were emphasized in Part 2.1. (3) Sputum liquefaction test results: the results of sputum liquefaction tests often include colonizing and non-pathogenic bacteria. To address this challenge, we established a panel of three independent hematology experts—two deputy chief physicians and one chief physician—who comprehensively evaluate respiratory infections and clinically relevant pathogens. This evaluation is based on general bacterial cultivation, blood culture, fungal culture, fungal smears, respiratory pathogen detection (including viruses), new coronavirus nucleic acid detection, 198-pathogen RTI tNGS panel results, imaging findings, patient history, clinical symptoms, and treatment response. The 198-pathogen RTI tNGS clearly demonstrates advantages over TMT, especially in developing countries. It can help reduce the public health burden, lower individual economic costs, and provide more accurate and rapid guidance for treatment. For hospitals without NGS machines (such as ours), samples can be sent to specialized third-party laboratories for testing. For hospitals with NGS capabilities, it is recommended to actively implement this technology to reduce patient hospitalization costs and provide better guidance for clinical treatment.

While the 198-pathogen RTI tNGS panel demonstrates promising diagnostic capabilities, there are some prospectives for improving this tool: (1) increased sensitivity: future versions of the panel could be optimized to detect a broader range of pathogens, including rare or atypical organisms, to improve diagnostic sensitivity and comprehensiveness. (2) Enhanced sample types: expanding the tool to include other sample types, such as blood or nasopharyngeal swabs, could increase its applicability, especially for patients who cannot provide sputum samples or have difficulty with sputum collection. (3) Automated data analysis: developing more sophisticated algorithms for data analysis could enhance the accuracy of pathogen identification, reduce manual interpretation errors, and streamline the diagnostic process. (4) Broader clinical validation: conducting larger-scale clinical trials across diverse patient populations would help validate the panel’s performance in real-world settings and ensure its robustness across different types of respiratory infections in hematology patients. By addressing these areas, the 198-pathogen RTI tNGS panel can be further refined, making it a more effective and versatile diagnostic tool for respiratory infections in hematology patients.

In conclusion, myelosuppressed hematology patients are unable to undergo bronchoalveolar lavage due to severe thrombocytopenia (platelet counts <20 × 10^9^/L). Severe thrombocytopenia is a contraindication for bronchoalveolar lavage (BAL). In patients with severe thrombocytopenia, the risk of bronchial bleeding is significantly elevated, including the potential for massive hemoptysis. None of the patients enrolled in our study underwent bronchoalveolar lavage to obtain bronchial specimens. The positive rate of routine microbial detection based on sputum is relatively low, whereas targeted next-generation sequencing (tNGS) based on sputum is more convenient, efficient, and cost-effective as it can detect a wide range of pathogens with high specificity and can detect common pathogen resistance genes. The study also highlighted the heterogeneity among the patients. The rapid TAT and high diagnostic efficiency of 198-pathogen RTI tNGS panel will significantly impact antibiotic therapy and prognosis in these patients. Therefore, tNGS holds great promise in meeting the clinical diagnostic and treatment needs for respiratory tract infections in myelosuppressed hematology patients, addressing practical clinical challenges and offering substantial clinical application prospects and guidance significance.

## Data Availability

The raw data supporting the conclusions of this article will be made available by the authors, without undue reservation.

## Supplementary material

Supplementary Table S1 198-pathogen RTI tNGS panel.

Supplementary Table S2 Distribution of pathogens in the study cohort and detection rate heterogeneity between 198-pathogen RTI tNGS panel and TMT.

Supplementary Table S3 Baseline characteristics of the 20 patients.

Supplementary Table S4 Prices for different items.

Supplementary Table S5 Pathogen detection by 198-pathogen RTI tNGS panel and TMT.
